# Divergent effects of interleukin-6 and ghrelin on remission and relapse in depression treatment

**DOI:** 10.1093/ijnp/pyag032

**Published:** 2026-06-22

**Authors:** Jae-Min Kim, Hee-Ju Kang, Ju-Wan Kim, Min Jhon, Ju-Yeon Lee, Sung-Wan Kim, Il-Seon Shin

**Affiliations:** Department of Psychiatry, Chonnam National University Medical School, Gwangju, Korea; Department of Psychiatry, Chonnam National University Medical School, Gwangju, Korea; Department of Psychiatry, Chonnam National University Medical School, Gwangju, Korea; Department of Psychiatry, Chonnam National University Medical School, Gwangju, Korea; Department of Psychiatry, Chonnam National University Medical School, Gwangju, Korea; Department of Psychiatry, Chonnam National University Medical School, Gwangju, Korea; Department of Psychiatry, Chonnam National University Medical School, Gwangju, Korea

**Keywords:** depression, interleukin-6, ghrelin, antidepressant, remission, relapse

## Abstract

**Background:**

This study was to explore how serum ghrelin levels modulate the relationship between interleukin-6 (IL-6) and outcomes of antidepressant treatment, specifically focusing on 12-week remission and 24-month relapse in patients with depressive disorders.

**Methods:**

This investigation involved the analysis of baseline serum levels of ghrelin and IL-6 among 1086 patients enrolled in a naturalistic study of stepwise antidepressant therapy. Remission was determined by a Hamilton Depression Rating Scale (HAMD) score of 7 or less at 12 weeks. Those who responded (HAMD score  <14) at this juncture were subsequently monitored for relapse (HAMD score  ≥14) quarterly over a 24-month period. The study employed logistic regression models, adjusted for various sociodemographic and clinical factors, to evaluate the interaction between these biomarkers and treatment outcomes.

**Results:**

Findings revealed that while serum ghrelin levels did not independently affect treatment outcomes, they significantly influenced the association between elevated IL-6 levels and the risks of non-remission at 12 weeks and relapse at 24 months. Specifically, high IL-6 levels correlated with poorer outcomes predominantly in the presence of lower ghrelin levels, with these interactions reaching statistical significance in relapse outcomes post-adjustment.

**Conclusion:**

The study highlights the complex interplay between IL-6 and ghrelin in shaping the efficacy of antidepressant treatments, demonstrating divergent influences on remission and relapse. These results emphasize the importance of incorporating multiple biomarkers into predictive models to tailor antidepressant strategies more effectively. Continued research is needed to dissect the underlying dynamics of these biomarker interactions.

## Teaser

1. Baseline ghrelin significantly modified the association between IL-6 and antidepressant treatment outcomes in depressive disorders. The adverse effect of elevated IL-6 was mainly observed in patients with lower ghrelin levels.
2. Higher IL-6 levels were associated with poorer short-term remission and greater long-term relapse risk, whereas ghrelin itself did not show an independent association with outcomes.
3. The IL-6 × ghrelin interaction remained significant for 24-month relapse after adjustment, suggesting that combined inflammatory and neuroendocrine profiling may improve prediction of depression course.

## Graphical abstract

**Graphical abstract:**
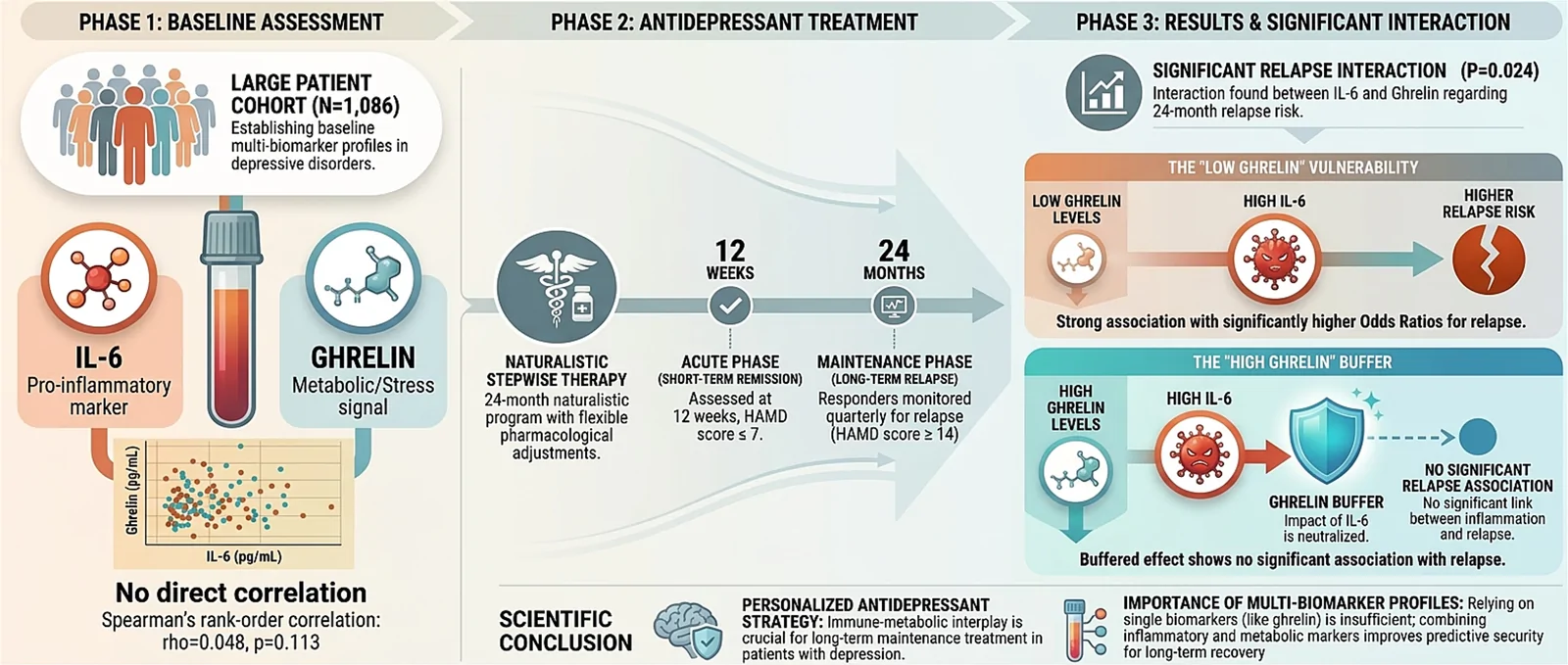

## Introduction

Depression continues to be one of the most prevalent psychiatric disorders globally, posing significant clinical challenges due to its complex pathophysiology and variable treatment outcomes.^1^^,^^2^ Despite advancements in therapeutic strategies, only about one-third of patients achieve remission during acute treatment phases.^3^ Moreover, over half of the individuals who experience a first depressive episode are likely to endure subsequent ones, with the likelihood of additional episodes increasing thereafter.^4^ This variability underscores the substantial burden of the disease and highlights the critical need for predictive biomarkers that can herald more personalized treatment approaches to enhance response rates and reduce relapse probabilities.^5^

Peripheral blood biomarkers are accessible and cost-effective, making them particularly advantageous for routine clinical monitoring. Among these, elevated inflammatory responses, as evidenced by increased levels of certain cytokines, have been consistently implicated in the treatment response and exacerbation of depressive symptoms.^6^ Interleukin-6 (IL-6) is one of the principal mediators in this inflammatory cascade, stimulating inflammatory and auto-immune processes in many diseases, including depression.^7^ Research into the role of IL-6 in antidepressant response has produced varied results; some studies report that higher baseline levels predict poorer outcomes,^8^ while others find no significant correlation^9^ or even contradictory results,^10^ as recent meta-analyses suggest.^11^ The role of IL-6 related to depression relapse has also been inconclusive, as most prospective studies have reported no specific associations between IL-6 levels and the recurrence of depression.^12–15^

These controversial results may stem from the complex interplay between IL-6 and other biomarkers.^16^ One such candidate is ghrelin, a hormone that is mainly produced and secreted by the fundus of the stomach, penetrates the blood–brain barrier, and exerts pleiotropic effects in the central nervous system.^17^ It has been reported that ghrelin regulates inflammatory reactions in various body systems and disease models.^18–20^ Notably, experimental studies have demonstrated that ghrelin can suppress the production of pro-inflammatory cytokines, including IL-6, suggesting a functional cross-talk between metabolic and inflammatory pathways.^21^ Moreover, evidence from non-psychiatric conditions, such as cardiovascular and metabolic disorders, indicates that ghrelin may counteract inflammation-driven pathophysiology.^22^ In addition, increased ghrelin levels were found in animal models of depression, interpreted as a stress coping mechanism.^23^^,^^24^ However, clinical studies exploring ghrelin's role in depressive disorders are limited, with few reporting alterations in ghrelin levels.^25^

Given IL-6's well-documented influence on inflammatory pathways implicated in depression and ghrelin's less explored potential protective antidepressant effects through modulation of stress responses, we hypothesize that ghrelin status may modulate the effects of IL-6 on antidepressant responses and relapse risk. This hypothesis remains untested, highlighting a significant gap in the current literature. Our study addresses this gap by utilizing a prospective cohort of Korean patients with depressive disorders undergoing structured antidepressant treatment, aiming to explore how baseline serum ghrelin levels affect the relationship between serum IL-6 and treatment outcomes, including remission at 12 weeks and relapse at 24 months.

## Methods

### Study framework

This study is an integral component of the broader MAKE Biomarker Discovery for Enhancing Antidepressant Treatment Effect and Response (MAKE BETTER) program. The foundational methodology for this investigation has been previously delineated^26^ and is registered at cris.nih.go.kr (identifier: KCT0001332). This project aimed to develop a treatment-response prediction index based on biopsychosocial markers for patients with depressive disorders. Recruitment specifically targeted a diverse cohort that included various depressive subtypes and comorbid physical illnesses, reflecting real-world clinical scenarios. Baseline data collection encompassed socio-demographic and clinical details, as well as a comprehensive panel of blood biomarkers, using structured clinical report forms completed by coordinators trained in psychiatric research. The treatment was administered in a naturalistic manner, allowing flexibility in the choice of medication, dosage, and treatment plans based on patient and clinician preferences, structured around predefined intervals and assessment points. Initial treatment consisted of 3-week antidepressant monotherapy, with possible adjustments at 3-week intervals during the first 12 weeks of the acute treatment phase, and subsequently at three-month intervals up to 24 months during the continuation and maintenance phases. Monitoring of participants was rigorously maintained at each interval—tri-weekly up to 12 weeks and tri-monthly up to 24 months—through structured clinical report forms completed by coordinators blinded to treatment specifics. From this prospective dataset, various hypotheses have been explored, examining how biochemical markers interact with other biomarkers or psychosocial factors to enhance the overall treatment-response prediction index.^27–29^ As detailed in the Introduction, this analysis specifically focuses on the associations between IL-6 and ghrelin levels and their impact on antidepressant treatment responses and relapse. The study protocols conformed to ethical standards sanctioned by the Institutional Review Board of Chonnam National University Hospital (CNUH 2012-014), adhering to all pertinent ethical guidelines.

### Participants

The study involved a cohort of individuals diagnosed with depressive disorders (DSM-IV-TR).^4^ Recruitment occurred at the outpatient psychiatric department of CNUH from March 2012 to April 2017. To ensure a representative sample, the study utilized inclusive eligibility and minimal exclusion criteria, further details of which are documented in the Supplementary Material. Each participant enrolled was beginning a new course of treatment, with antidepressants initiated for either first-time or recurrent depressive episodes, and had not received antidepressant treatment for at least four weeks prior to study entry. The primary aim of the MAKE BETTER study was to identify predictive biomarkers for outcomes of anti-depressive treatment. Accordingly, all participants were treated exclusively with anti-depressive pharmacotherapy, having provided informed consent after receiving thorough documentation about the study.

### Serum biomarkers

To ensure standardized conditions for blood sample collection, participants were required to fast overnight but were permitted to drink water. Before undergoing venipuncture, they were also asked to rest quietly for 25 to 45 minutes to minimize stress and stabilize physiological responses. The serum level of IL-6 was determined using a Human High Sensitivity T Cell Magnetic Bead Panel (EMD Millipore, Billerica, USA). Simultaneously, serum ghrelin levels were quantified using a GHRELIN (Total) radioimmunoassay kit (EMD Millipore, MO, USA). These assays were performed at the Global Clinical Central Lab in Yongin, Korea. In the absence of established clinical cutoffs for serum IL-6 and ghrelin levels, both biomarkers were primarily analyzed as continuous variables to preserve statistical power and capture potential dose–response relationships. To facilitate interpretation and comparison with prior exploratory biomarker studies, additional categorical analyses were performed by dichotomizing each biomarker into “high” and “low” groups based on the median value, allowing for an equal distribution of participants between groups. The median-split method is frequently employed in exploratory biomarker research to aid in the identification of potential associations and interactions and to generate hypotheses for further investigation.^30^

### Baseline covariates

Detailed sociodemographic data were collected for each participant, covering age, sex, years of educational attainment, marital status (married or unmarried), living situation (alone or with others), employment status, and monthly income (categorized as either above or below $2000 USD). Clinical information recorded included the diagnosis of depressive disorders according to previously specified criteria, with additional details on melancholic and atypical features. The clinical profile also included age at depression onset, total illness duration, number of depressive episodes, duration of the current episode, family history of depression, and the presence of comorbid physical conditions, which were evaluated using a comprehensive health questionnaire that covered 15 different systems. Additional baseline evaluations encompassed body mass index (BMI) and smoking status. To assess symptoms, several scales were employed: the Hospital Anxiety and Depression Scale for depression and anxiety (HADS-D and HADS-A),^31^ the Social and Occupational Functioning Assessment Scale (SOFAS),^32^ and the Alcohol Use Disorder Identification Test (AUDIT).^33^ These tools provided quantitative measures of symptom severity in various domains.

### Stepwise pharmacotherapy

The details of the treatment protocol, previously outlined in our publications,^26^^,^^34^ are briefly summarized in the Supplementary Material. Initially, the therapeutic regimen began with a period of monotherapy. Based on the individual patient’s response to treatment and tolerance of side effects, which were evaluated tri-weekly during the first 12 weeks and subsequently tri-monthly up to 24 months, adjustments to the pharmacological strategy were made as needed. These adjustments could include switching medications, augmenting with additional agents, or employing a combination of multiple drugs, all within a flexible and adaptive treatment framework.

### Outcomes

#### Remission

Remission was assessed using the Hamilton Depression Rating Scale,^35^ with a score of 7 or less defining remission. Although remission status was evaluated at 3, 6, 9, and 12 weeks, participants were classified as remitted based on their status at the 12-week assessment. Those who met the remission criterion at 12 weeks were categorized as remitted, regardless of their remission status at earlier time points.

#### Relapse

For relapse analysis, patients who achieved a response in the initial 12-week acute treatment phase with a HAMD score of less than 14 (distinct from the remission threshold) were examined. Relapse monitoring commenced from the 12-week evaluation and continued tri-monthly up to 24 months. A relapse was defined as a HAMD score of 14 or higher at any point during the follow-up period, consistent with definitions from previous research.^36^^,^^37^ This threshold was selected to represent a clinically meaningful return of depressive symptoms, consistent with prior longitudinal depression studies.

### Statistical analysis

Baseline characteristics of patients were stratified and compared across four groups defined by the median levels of IL-6 and ghrelin: higher ghrelin/low IL-6, high ghrelin/high IL-6, low ghrelin/low IL-6, and low ghrelin/high IL-6. These comparisons were made using analysis of variance (ANOVA) or chi-square tests as appropriate, with Scheffe’s post-hoc tests following ANOVA and specified pairwise comparisons for chi-square when necessary. For remission analysis, these characteristics were evaluated by remission status (remission vs. no remission) using independent t-tests or chi-square tests, depending on the nature of the data. Similarly, for the analysis of relapse, characteristics along with treatment details were compared by relapse status (relapse vs. no relapse) using the same statistical tests. The selection of covariates for further analyses in adjusted models was based on their statistical significance (*P* < .05) and the absence of collinearity. In addition, sensitivity analyses were conducted using a pre-specified set of clinically relevant covariates selected a priori based on clinical relevance and prior literature, including demographic factors, baseline symptom severity, illness characteristics, and lifestyle factors.^36^^,^^37^ The relationships between serum IL-6 and ghrelin levels were assessed with Spearman’s rank-order correlation. The effects of individual serum IL-6 and ghrelin levels on the likelihood of achieving 12-week remission and experiencing 24-month relapse were examined through logistic regression, both in unadjusted and adjusted models. The potential interactive effects of serum IL-6 and ghrelin levels on remission and relapse were primarily examined using multinomial logistic regression models with biomarkers treated as continuous variables. The main models were adjusted for covariates identified in the primary analysis based on statistical significance and absence of collinearity. To evaluate the robustness of the findings, additional sensitivity analyses were conducted. For remission outcomes, sensitivity analyses included adjustment using a pre-specified set of clinically relevant covariates and analyses using log-transformed biomarker values. For relapse outcomes, additional sensitivity analyses further included inverse probability weighting to account for non-random attrition and stratified analyses according to treatment strategy (monotherapy vs augmentation/combination), in addition to the pre-specified covariate adjustment and log-transformed biomarker analyses. To facilitate visualization and interpretation of the interaction effects, supplementary categorical analyses were also performed. Specifically, both biomarkers were first dichotomized using median values, and additional analyses were conducted by categorizing IL-6 into tertiles while retaining ghrelin as a binary variable, to explore potential non-linear patterns and ensure robustness of the observed interaction. All statistical procedures were conducted as two-tailed tests with a significance threshold maintained at *P* < .05. These analyses were performed using IBM SPSS Statistics, Version 27.

## Results

### Recruitment process and probabilities of remission and relapse

The recruitment process and the probabilities of remission and relapse are depicted in Figure 1. Initially, 1262 individuals were assessed at the commencement of the study. Of these, 1094 (86.7%) consented to provide blood samples, and 1086 (86.1%) were followed up for at least one follow-up during the 12-week treatment period, forming the cohort for remission analysis. Remission was achieved by 490 (45.1%) of these patients. A comparative analysis of the 1086 participants included in the study and the 176 who were not included revealed no significant differences in baseline characteristics between the two groups (all *P*-values > .1). By the conclusion of the 12 weeks, 263 patients (24.2%) had a HAMD score of 14 or higher, while 823 maintained scores below 14. Of those, 710 (86.3%) were monitored throughout the 24-month follow-up period, constituting the cohort for relapse analysis. Relapse was observed in 301 (42.4%) of these individuals. Dropout at 24 months was significantly associated with unemployment and lower SOFAS scores at baseline.

**Figure 1:**
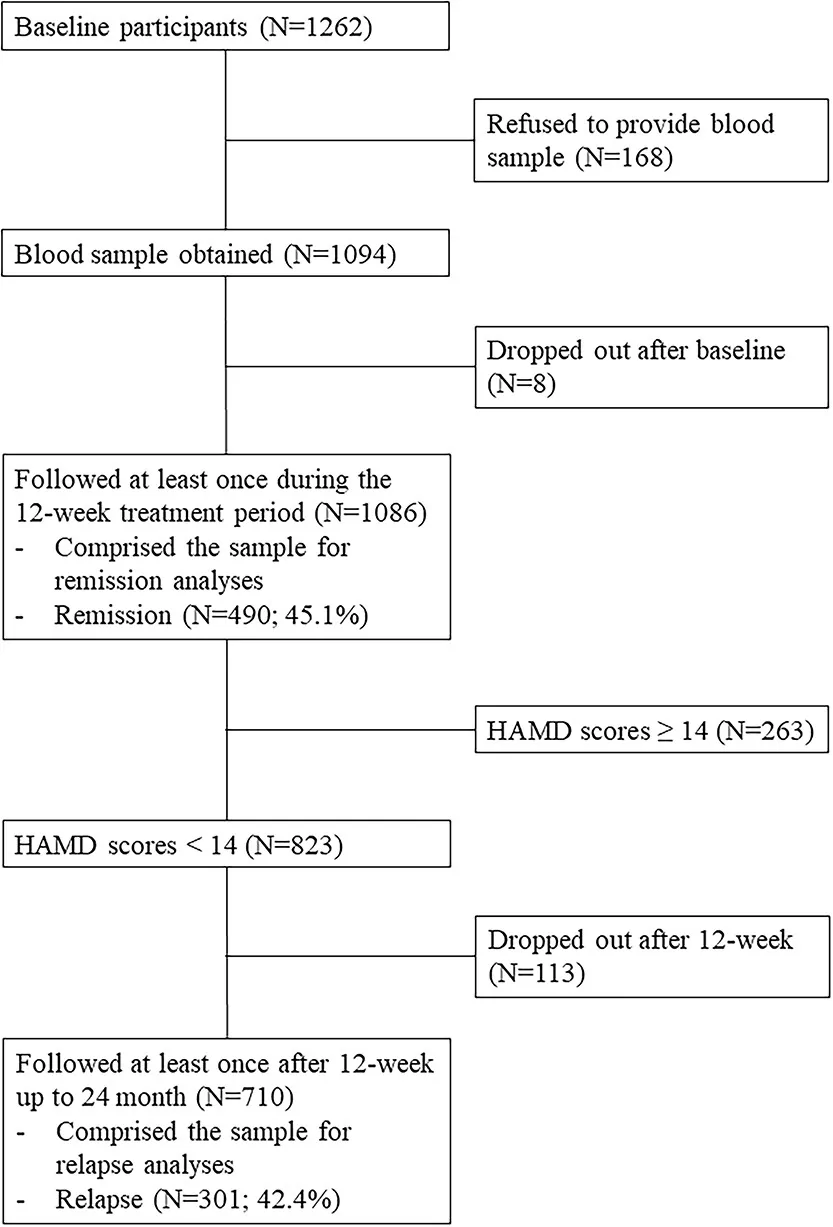
Participants flow and treatment outcomes status.

### Baseline characteristics

The distribution of baseline characteristics, stratified by median serum levels of IL-6 (1.65 pg/mL) and ghrelin (378.5 pg/mL), is presented in Table 1. Notable differences among these groups were observed in variables such as age, sex, and age at onset. Further explorations of these baseline characteristics in relation to remission and relapse are provided in Supplementary Tables S1 and S2. Factors significantly associated with a non-remission outcome included younger age, lower income, earlier age of onset, longer duration of the current depressive episode, non-smoking status, higher scores on the HADS-D and HADS-A, and lower scores on the SOFAS. Significant predictors of relapse encompassed younger age at onset, higher initial scores on HADS-D and HADS-A, lower SOFAS scores, a higher number of treatment steps, and poorer medication adherence. Based on their statistical significance (*P* < .05) and role in controlling potential multicollinearity, seven variables—age, sex, monthly income, number of depressive episodes, current smoking, and scores on HADS-A and SOFAS—were selected for remission analysis. Nine variables, including the aforementioned seven plus treatment steps and medication adherence after 12 weeks, were chosen for relapse analysis. Additionally, the correlation analysis revealed no significant relationship between baseline serum levels of IL-6 and ghrelin (rho = 0.048; *p*-value = .113).

**Table 1:** Baseline characteristics according to baseline serum levels of interleukin-6 (IL-6) and ghrelin in patients with depressive disorders (N = 1086).

		Higher ghrelin		Lower ghrelin		Statistical coefficients for group differences^a^	Post-hoc comparisons (*P* < .05)
		Lower IL-6 (N = 264): 1	Higher IL-6 (N = 273): 2	Lower IL-6 (N = 276): 3	Higher IL-6 (N = 273): 4		
**Socio-demographic characteristics**							
Age, mean (SD) years		57.5 (14.3)	55.0 (15.6)	59.9 (13.1)	55.3 (15.9)	F = 6.407 *P* < .001	1,4 < 3
Sex, N (%) female		188 (71.2)	208 (76.2)	168 (60.9)	181 (66.3)	χ^2^ = 16.466 *P* = .001	1,2 > 3; 2 > 4
Education, mean (SD) years		8.9 (4.7)	9.3 (4.8)	8.6 (4.8)	9.6 (4.8)	F = 2.026 *P* = .109	n-s
Marital status, N (%) unmarried		85 (32.2)	81 (29.7)	75 (27.2)	85 (31.1)	χ^2^ = 1.837 *P* = .607	n-s
Living alone, N (%)		45 (17.0)	37 (13.6)	45 (16.3)	40 (14.7)	χ^2^ = 1.555 *P* = .670	n-s
Unemployed status, N (%)		73 (27.7)	77 (28.2)	83 (30.1)	83 (30.4)	χ^2^ = 0.726 *P* = .867	n-s
Monthly income, N (%) <2000 USD		166 (62.9)	160 (58.6)	169 (61.2)	153 (56.0)	χ^2^ = 3.029 *P* = .387	n-s
**Clinical characteristics**							
Major depressive disorder, N (%)		231 (87.5)	238 (87.2)	233 (84.4)	223 (81.7)	χ^2^ = 4.757 *P* = .191	n-s
Melancholic feature, N (%)		31 (11.7)	41 (15.0)	51 (18.5)	39 (14.3)	χ^2^ = 4.942 *P* = .176	n-s
Atypical feature, N (%)		16 (6.1)	25 (9.2)	13 (4.7)	15 (5.5)	χ^2^ = 5.237 *P* = .155	n-s
Age at onset, mean (SD) years		51.1 (16.7)	48.8 (16.9)	54.6 (15.1)	49.1 (16.8)	F = 7.169 *P* < .001	1,4 < 3
Duration of illness, mean (SD) years		6.6 (10.0)	6.5 (9.5)	5.6 (8.4)	6.5 (9.3)	F = 0.667 *P* = .572	n-s
Number of depressive episodes, mean (SD)		2.4 (5.4)	2.1 (4.3)	2.2 (5.0)	2.4 (5.4)	F = 0.251 *P* = .861	n-s
Duration of present episode, mean (SD) months		8.3 (12.1)	6.8 (9.1)	6.7 (9.1)	7.8 (11.1)	F = 1.525 *P* = .206	n-s
Family history of depression, N (%)		37 (14.0)	44 (16.1)	39 (14.1)	38 (14.0)	χ^2^ = 0.726 *P* = .867	n-s
Number of physical disorders, mean (SD)		1.5 (0.5)	1.5 (0.5)	1.5 (0.5)	1.5 (0.5)	F = 1.317 *P* = .267	n-s
Body mass index, mean (SD) kg/m^2^		23.1 (3.0)	23.1 (3.3)	23.2 (3.0)	23.4 (3.3)	F = 0.701 *P* = .552	n-s
Current smoking, N (%)		38 (14.4)	25 (9.2)	34 (12.3)	26 (9.5)	χ^2^ = 4.906 *P* = .179	n-s
**Assessment scales, mean (SD) scores**							
Hospital Anxiety & Depression Scale-depression subscale		13.4 (3.9)	13.4 (4.0)	14.1 (3.9)	13.9 (3.9)	F = 1.950 *P* = .120	n-s
Hospital Anxiety & Depression Scale-anxiety subscale		11.5 (4.0)	11.4 (4.3)	12.2 (3.9)	12.1 (3.9)	F = 2.466 *P* = .061	n-s
Social and Occupational Functioning Assessment Scale		56.7 (7.5)	55.8 (7.2)	56.1 (7.4)	55.2 (7.7)	F = 2.042 *P* = .106	n-s
Alcohol Use Disorder Identification Test		5.8 (9.1)	4.7 (8.1)	5.8 (9.6)	5.1 (8.7)	F = 1.038 *P* = .375	n-s

a Analysis of variance test or χ^2^ test, as appropriate.

### Individual effects of the serum IL-6 and ghrelin levels on remission and relapse

The relationships between baseline serum IL-6 and ghrelin levels with remission and relapse outcomes are depicted in Table 2. Elevated IL-6 levels, analyzed both as continuous and categorical variables, were significantly associated with a lack of remission in both unadjusted and adjusted models. Conversely, serum ghrelin levels, examined as either continuous categories or binarily, showed no significant influence on remission status under any adjustment conditions. Similarly, neither serum IL-6 nor ghrelin levels demonstrated significant associations with relapse outcomes, regardless of how they were categorized or adjusted.

**Table 2:** Individual effects of serum interleukin-6 (IL-6) and ghrelin levels as binary and continuous variables on remission and relapse status.

Exposure	Group	Remission analyses (N = 1086)					Relapse analyses (N = 710)			
		N	No. (%)	OR (95% CI)			N	No. (%)	OR (95% CI)	
				Unadjusted	Adjusted^a^				Unadjusted	Adjusted^b^
IL-6 (increasing)	N/A	1086	N/A	0.97 (0.94-0.99)^*^	0.97 (0.95-1.00)^*^		710	N/A	1.01 (0.98-1.05)	1.03 (0.89-1.21)
IL-6 (binary)	Lower	540	263 (48.7)	Ref	Ref		359	148 (41.2)	Ref	Ref
	Higher	546	227 (41.6)	0.75 (0.59-0.95)^*^	0.88 (0.77-0.99)^*^		351	153 (43.6)	1.10 (0.82-1.48)	1.14 (0.85-1.53)
										
Ghrelin (decreasing)	N/A	1086	N/A	1.00 (0.99-1.00)	1.00 (0.99-1.00)		710	N/A	1.00 (1.00-1.01)	1.00 (1.00-1.01)
Ghrelin (binary)	Higher	543	258 (47.5)	Reference	Reference		355	146 (41.1)	Reference	Reference
	Lower	543	232 (42.7)	0.83 (0.65-1.05)	0.82 (0.64-1.05)		355	155 (43.7)	1.20 (0.89-1.62)	1.14 (0.84-1.55)

a Adjusted for age, sex, monthly income, number of depressive episodes, current smoking, and scores on Hospital Anxiety & Depression Scale-anxiety subscale and Social and Occupational Functional Assessment Scale.

b Adjusted for ^a^ plus treatment step after 12 week and poor medication adherence after 12 week.

^*^ *P* < .05.

### Modulating effects of ghrelin on the associations of IL-6 with remission

In the primary analysis using continuous biomarker values, the interaction between IL-6 and ghrelin was not significantly associated with 12-week remission (Wald = 1.239, *P* = .266) (Table S3). This non-significant finding remained consistent across sensitivity analyses, including adjustment using a pre-specified set of clinically relevant covariates (as detailed in the footnotes of Table S3) (Wald = 1.242, *P* = .261) and analyses using log-transformed biomarker values (Wald = 1.373, *P* = .243). Categorical analyses showed a similar pattern, with higher IL-6 levels being associated with non-remission primarily among participants with lower ghrelin levels; however, the interaction terms did not reach statistical significance (Figure 2). Additional analyses using tertiles of IL-6 yielded comparable patterns without demonstrating statistically significant interaction effects (Supplementary Figure S1).

**Figure 2:**
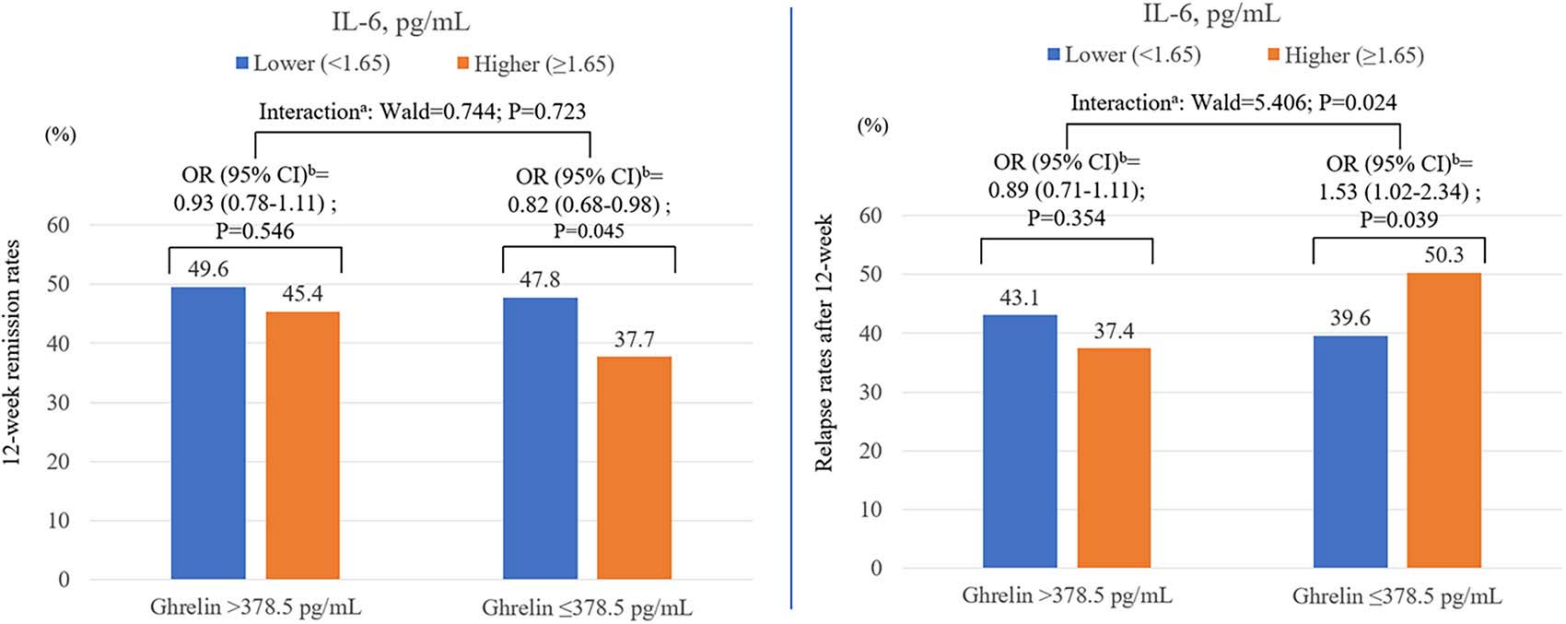
Modulating effects of serum ghrelin on the associations of interleukin-6 (IL-6) with remission and relapse status. ^a^Interactive modulating effects of ghrelin on the associations of IL-6 with remission and relapse status were estimated using multinomial logistic regression; and ^b^direct effects of IL-6 within each ghrelin stratum, expressed as odds ratios (95% confidence intervals) [OR (95% CI)], were calculated using binary logistic regression for lower (<1.65 pg/mL) vs. higher (≥1.65 pg/mL) IL-6 levels on remission and relapse status, after adjustment for age, sex, monthly income, number of depressive episodes, current smoking, and scores on hospital anxiety & depression scale-anxiety subscale and social and occupational functional assessment scale for remission analyses; plus treatment step after 12 week and poor medication adherence after 12 week for relapse analyses. ^*^P < 0.05.

### Modulating effects of ghrelin on the associations of IL-6 with relapse

In contrast, in the primary analysis using continuous biomarker values, the interaction between IL-6 and ghrelin was significantly associated with 24-month relapse (Wald = 5.984, *P* = .018) (Table 3). This finding remained robust across multiple sensitivity analyses. The interaction was slightly strengthened when using log-transformed biomarker values (Wald = 6.813, *P* = .009), while remaining largely consistent after adjustment using a pre-specified set of clinically relevant covariates (as detailed in the footnotes of Table 3) (Wald = 6.030, *P* = .017). Furthermore, the interaction remained statistically significant after accounting for potential bias due to non-random attrition using inverse probability weighting (Wald = 5.679, *P* = .024). In stratified analyses by treatment strategy, a similar pattern was observed, with the interaction remaining significant in the augmentation/combination group (Wald = 6.732, *P* = .011) but not in the monotherapy group (Wald = 3.123, *P* = .277). Categorical analyses further supported these findings, showing that higher IL-6 levels were associated with increased relapse risk predominantly among participants with lower ghrelin levels, whereas no such association was observed among those with higher ghrelin levels (Figure 2). Additional analyses using tertiles of IL-6 demonstrated a consistent pattern, with slightly stronger statistical significance of the interaction (Supplementary Figure S1).

**Table 3:** Interactive effects of continuous serum interleukin-6 (IL-6) and ghrelin levels on relapse: primary and sensitivity analyses across alternative modeling strategies.

Model		N	Interaction OR (95% CI)	Wald	*p*-value
1 (primary): Continuous interaction adjusted for covariates identified in the main analysis		710	1.01 (1.00-1.01)	5.984	.018
2: Adjusted for a pre-specified set of clinically relevant covariates		710	1.01 (1.00-1.01)	6.030	.017
3: Log-transformed biomarkers		710	1.01 (1.00-1.01)	6.813	.009
4: Inverse probability weighting for attrition		823	1.00 (1.00-1.01)	5.679	.024
5: Stratified by treatment strategy	Monotherapy	332	1.00 (0.99-1.01)	3.123	.277
	Augmentation/combination	378	1.01 (1.00-1.02)	6.732	.011

Models 1, 3, 4, and 5 were adjusted for age, sex, monthly income, number of depressive episodes, current smoking, scores on the Hospital Anxiety and Depression Scale–anxiety subscale and the Social and Occupational Functioning Assessment Scale, and treatment step after 12 week and poor medication adherence after 12 week.

Model 2 was adjusted for a pre-specified set of clinically relevant covariates, including age, sex, number of depressive episodes, duration of the current episode, number of physical disorders, body mass index, current smoking, and scores on the Hospital Anxiety and Depression Scale–depression subscale.

## Discussion

In our study, which employed a naturalistic and prospective design to mirror real-world clinical conditions, we discovered that serum ghrelin levels, while not directly influencing treatment outcomes, moderated the impact of serum IL-6 levels on the likelihood of 12-week non-remission and 24-month relapse. Specifically, elevated IL-6 levels were associated with adverse outcomes predominantly in the context of lower ghrelin levels, whereas such associations were not observed when ghrelin levels were higher. Importantly, these findings were consistently observed across multiple analytical approaches. The interaction between IL-6 and ghrelin remained evident in the primary analyses using continuous biomarker values, as well as in sensitivity analyses employing alternative covariate adjustments and log-transformed variables. Complementary categorical analyses, including both median-based stratification and tertile-based categorization, demonstrated similar patterns. Notably, the interaction effect reached statistical significance for the relapse outcome, even in the absence of a direct correlation between IL-6 and ghrelin, and after adjustment for relevant covariates.

The differential impacts of IL-6 on antidepressant treatment outcomes between states of higher and lower ghrelin can be elucidated by several mechanisms. Primarily, ghrelin possesses anti-inflammatory properties that may counterbalance the pro-inflammatory effects of cytokines such as IL-6.^18–20^ In scenarios where ghrelin levels are elevated, there could be a shift towards an anti-inflammatory or neutral state, diminishing the potential for IL-6 to induce depressive symptoms via inflammatory pathways. Conversely, reduced levels of ghrelin might allow the unchecked inflammatory activity of IL-6 to exacerbate depressive symptoms or diminish the efficacy of antidepressants. The significant modulatory effects of IL-6 and ghrelin on treatment outcomes, despite their lack of direct baseline correlation, suggest complex biochemical interactions influenced by depression management. These effects may become apparent under specific physiological conditions, such as those induced by antidepressant treatment, which could modulate receptor sensitivities or protein expression levels, thus altering the interactions between IL-6 and ghrelin during treatment.^38^

Notably, the interaction between IL-6 and ghrelin on relapse was statistically significant only among patients receiving augmentation or combination treatment, but not among those treated with monotherapy. This finding may be interpreted in the context of treatment complexity. Patients requiring augmentation or combination strategies are likely to represent a subgroup with more severe or treatment-resistant depression, in whom biological vulnerabilities—including inflammatory and stress-related mechanisms—may play a more prominent role.^39^

Additionally, ghrelin's regulatory influence on the hypothalamic–pituitary–adrenal (HPA) axis plays a vital role in stress response and mood regulation.^17^ Elevated ghrelin levels may stabilize the HPA axis, reducing vulnerability to stress-induced exacerbation of depressive symptoms, potentially aggravated by high IL-6 levels. When ghrelin levels are low, the absence of this stabilizing effect permits the pro-inflammatory actions of IL-6 to more significantly influence depressive outcomes. Furthermore, ghrelin's role in regulating metabolism and energy homeostasis is critical for maintaining overall brain health and functionality.^40^ Given that metabolic dysregulation is a frequent complication in depression, the modulation of energy balance by high ghrelin levels could confer resilience against metabolic disturbances typically associated with elevated IL-6 levels. These insights underscore the intricate mechanisms through which these biomarkers influence depression treatment outcomes, highlighting their importance in understanding and managing depression despite their lack of direct baseline correlation.

The observation that ghrelin's modulating effect on IL-6 demonstrated a significant interaction for 24-month relapse, but not for 12-week remission, suggests that the influence of these biomarkers may be more pronounced or accumulative over extended periods. This distinction between short-term and long-term effects can be explained through several potential mechanisms. Firstly, chronic exposure to elevated IL-6 levels, particularly when not counterbalanced by adequate ghrelin levels, may lead to sustained inflammatory responses and neuroplastic changes. These changes can have cumulative detrimental effects on brain function and mood regulation,^6^ increasing the likelihood of relapse over time—a process not as discernible during the shorter duration assessed for remission. Secondly, over longer periods, the cumulative effects of behavioral and psychological responses to depression, such as learned helplessness, coping styles, or lifestyle changes, might more dynamically interact with biological factors like IL-6 and ghrelin.^41^ These interactions, while subtle in the acute phase of treatment, become significant predictors of long-term outcomes such as relapse. Additionally, factors like treatment adherence, as observed in our study (see Supplementary Table S2), may influence outcomes such as relapse. Importantly, including this as one of the covariates did not alter the findings, underscoring the robustness of the observed interactions between ghrelin, IL-6, and depression relapse over the long term.

With respect to individual associations, as noted in the Introduction, the relationship between IL-6 and outcomes such as remission or relapse in depression has been highly controversial. Our study offers a partial explanation for this controversy by incorporating ghrelin levels into our analysis. Specifically, while isolated IL-6 levels have been linked to varied effects on depression outcomes, introducing ghrelin reveals a modulatory capacity that helps clarify these inconsistent findings. On one hand, prior research into the direct impact of ghrelin levels on antidepressant treatment outcomes has been limited. Our results suggest that although ghrelin alone does not significantly influence these outcomes, it has the potential to modulate the impacts of other biomarkers like IL-6. This insight underscores the importance of considering interactions between multiple biomarkers rather than focusing solely on their individual effects,^42^ highlighting the need for further research in this area to fully understand the complex biochemical interactions affecting depression treatment.

Our research is subject to several limitations. First, biomarker levels were measured only at baseline, limiting our ability to observe how changes in these markers due to treatment might influence treatment responses—an effect previously shown to be significant by Basterzi et al.^10^ Additionally, our study's naturalistic design, while mirroring real-world clinical settings, does not employ a uniform treatment protocol, which introduces variability in treatment outcomes. The application of various antidepressants within a stepwise pharmacotherapy framework also complicates the precise identification of how inflammatory biomarkers predict treatment responses, as such variability could obscure or enhance the markers' predictive accuracy depending on the treatment scenario. Moreover, depressive disorders represent a highly heterogeneous condition, encompassing diverse clinical phenotypes with varying severity, course, and underlying biological mechanisms. This heterogeneity may have influenced the observed associations, as biomarker effects could differ across subgroups of patients, potentially contributing to variability in treatment outcomes. In addition, relapse analyses included participants who achieved treatment response (HAMD <14), rather than restricting the sample to those in full remission (HAMD ≤7). Given that residual depressive symptoms are known to increase the risk of relapse, this broader inclusion criterion may have influenced the observed associations. However, this approach was adopted to better reflect real-world clinical practice and to retain sufficient statistical power. Furthermore, attrition during the 24-month follow-up was not random, as dropout was associated with unemployment and lower SOFAS scores, suggesting that the analyzed sample may have been biased toward higher-functioning individuals. This may have led to a potential overestimation of the observed associations. However, sensitivity analyses using inverse probability weighting to account for differential follow-up yielded similar results, with the interaction between IL-6 and ghrelin on relapse remaining statistically significant, supporting the robustness of our findings. Finally, as a single-center study, the generalizability of our results may be constrained, potentially not reflecting the variety of patient populations seen in more extensive clinical environments.

The study has several strengths that enhance its scientific contribution. A key advantage is the substantial sample size employed, which affords robust statistical power and marks a significant improvement over many previous studies that have examined biomarkers in antidepressant treatment outcomes. This allows for more conclusive findings. Additionally, high follow-up rates ensure that data collection is thorough and attrition rates are low, bolstering the reliability of our results and mitigating the impact of potential biases that often arise from incomplete data sets. Moreover, our research is innovative in exploring the combined effects of serum IL-6 and ghrelin on antidepressant treatment responses. This approach offers new perspectives on the complex interactions between inflammatory markers and other systemic markers, enhancing our understanding of their roles. These novel insights help address persistent controversies in the field, particularly around the role of IL-6 in antidepressant treatment outcomes, a topic previously discussed in meta-analytical studies such as those by Liu et al.^11^ Overall, our findings contribute significantly to the body of knowledge, potentially guiding future research and clinical practice.

In conclusion, our study has elucidated the divergent effects of IL-6 and ghrelin on remission and relapse in depression treatment, underscoring the complex interplay between inflammatory and metabolic processes in shaping the trajectory of depression treatment outcomes. This finding emphasizes the need to consider both types of biomarkers when predicting outcomes in depression treatment, suggesting that a multifaceted approach may be more effective in managing and understanding this condition. The insights from our study have important clinical implications. By demonstrating that the effects of IL-6 on depression treatment outcomes can be modulated by ghrelin levels, our findings suggest that assessing both inflammatory and metabolic markers prior to treatment may improve stratification and prognostic evaluation. However, given the observational nature of our findings and the absence of a direct effect of ghrelin on outcomes, these results should not be interpreted as evidence for therapeutic targeting of ghrelin. Rather, they support the hypothesis that combined biomarker profiles may help identify patients at higher risk of poor outcomes, including relapse. Future research should focus on longitudinal studies that track changes in IL-6 and ghrelin levels throughout the course of treatment to better understand their dynamic interrelations and impact on treatment outcomes. Additionally, further studies are needed to clarify whether these biomarkers play a causal role or primarily reflect underlying pathophysiological processes. Investigating how these findings translate across different populations and settings will also be crucial in validating and refining the predictive value of IL-6 and ghrelin as biomarkers for depression treatment responses. Such efforts may ultimately contribute to the development of more personalized and biologically informed approaches to the management of depression.

## Supplementary Material

IL6_Ghr_RemRelap_04_Suppl_R_pyag032

## Data Availability

The data that support the findings of study are available from the corresponding author (J-M Kim) upon reasonable request.

## Significance statement

This study demonstrates that the interplay between interleukin-6 and ghrelin has divergent effects on remission and relapse in depression treatment. By identifying how low ghrelin amplifies the detrimental impact of elevated IL-6, our findings highlight the need to consider multi-biomarker profiles for predicting long-term treatment trajectories. These results suggest that incorporating inflammatory and neuroendocrine markers into routine assessment may improve personalized treatment strategies in clinical practice.
