# Supplementary material for: Divergent effects of interleukin-6 and ghrelin on remission and relapse in depression treatment
Source: Int J Neuropsychopharmacol. 2026 Jun 22;29(10):pyag032. doi: 10.1093/ijnp/pyag032 (PMC13688350; doi:10.1093/ijnp/pyag032)
Supplement: Supplementary file 1 [file il6_ghr_remrelap_04_suppl_r_pyag032.docx]

**Supplementary Material**

**Eligibility criteria**

Inclusion criteria were: i) aged older than 7 years; ii) diagnosed with MDD, dysthymic disorder, or depressive disorder not otherwise specified (NOS), using the Mini-International Neuropsychiatric Interview (MINI),^1^ a diagnostic psychiatric interview applying Diagnostic and Statistical Manual of Mental Disorders, Fourth Edition (DSM-IV) criteria^2^; iii) Hamilton Depression Rating Scale (HAMD)^3^ score ≥ 14; iv) able to complete questionnaires, understand the objective of the study, and sign the informed consent form. Exclusion criteria were: i) an unstable or uncontrolled medical condition; ii) unable to complete the psychiatric assessment or comply with the medication regimen, due to a severe physical illness; iii) current or lifetime DSM-IV diagnosis of bipolar disorder, schizophrenia, schizoaffective disorder, schizophreniform disorder, psychotic disorder NOS, or other psychotic disorder; iv) history of organic psychosis, dementia, epilepsy, or seizure disorder; v) history of anticonvulsant treatment; vi) hospitalization for any psychiatric diagnosis apart from depressive disorder (e.g., alcohol/drug dependence); vii) electroconvulsive therapy received for the current depressive episode; viii) pregnant or breastfeeding.

**Stepwise Pharmacotherapy**

Before treatment commencement, a comprehensive review was made of the patients’ clinical manifestations (e.g., psychotic and anxiety symptoms), severity of illness, physical comorbidities and medication profiles, and history of previous treatments. Minimal and maximal dosages of pharmacological agents were determined considering existing treatment guidelines.^4,5^ In the first treatment Step 1, patients received antidepressant treatment, taking into consideration these data and treatment guidelines,^5-7^ for 3 weeks. Antidepressants used were bupropion, desvenlafaxine, duloxetine, escitalopram, fluoxetine, mirtazapine, paroxetine, sertraline, venlafaxine, and vortioxetine. After Step 1 antidepressant monotherapy, next step pharmacotherapy could be administered every 3 weeks during the acute treatment phase (3, 6, 9, and 12 weeks with a 3-day allowable window), whenever needed. At the end of each step, overall effectiveness and tolerability were reviewed for proceeding with measurement-based next-step treatments. In cases of insufficient improvement (a HAMD score reduction of <30% from the baseline) or intolerable side effects, patients were instructed to choose whether they would prefer to remain in the current step or enter the next step strategies with switching (S), augmentation (A), combination (C), S + A, S + C, A + C, and S + A + C treatment. Patients were also allowed to receive next step treatment if they showed sufficient improvement (a HAMD score reduction of ≥ 30% from the baseline) and absent/tolerable side effects. For determining treatment strategies, each patient’s preference was given priority to maximize medication compliance and treatment outcomes.^8^ Antidepressants switched or combined were bupropion, desvenlafaxine, duloxetine, escitalopram, fluoxetine, mirtazapine, paroxetine, sertraline, venlafaxine, and vortioxetine. Augmented drugs were buspirone, lithium, triiodothyronine, and atypical antipsychotics including aripiprazole, risperidone, olanzapine, quetiapine, and ziprasidone.

**References**

1. Sheechan DV, Lecrubier Y, Sheehan KH, et al. The Mini- International Neuropsychiatric Interview (M.I.N.I.): the development and validation of a structured diagnostic psychiatric interview for DSM-IV and ICD-10. J Clin Psychiatry 1998;**59**(s):22-23
2. American Psychiatric Association. Diagnostic and Statistical Manual of Mental Disorders. 4th ed. American Psychiatric Press, 1994.
3. Hamilton M. A rating scale for depression. J Neurol Neurosurg Psychiatry 1960;**23**(1): 56-62. https://doi.org/10.1136/jnnp.23.1.56
4. Anderson IM, Ferrier IN, Baldwin RC, et al. Evidence-based guidelines for treating depressive disorders with antidepressants: a revision of the 2000 British Association for Psychopharmacology guidelines. J Psychopharmacol 2008;**22** (4): 343-396. https://doi.org/10.1177/0269881107088441.
5. Bauer M, Pfennig A, Severus E, et al. World Federation of Societies of Biological Psychiatry (WFSBP) guidelines for biological treatment of unipolar depressive disorders, part 1: update 2013 on the acute and continuation treatment of unipolar depressive disorders. World J Biol Psychiatry 2013;**14**:334-385. https://doi.org/ 10.3109/15622975.2013.804195.
6. Kennedy SH, Lam RW, McIntyre RS, et al. Canadian Network for Mood and Anxiety Treatments (CANMAT) 2016 Clinical Guidelines for the Management of Adults with Major Depressive Disorder: Section 3. Pharmacological Treatments. Can J Psychiatry 2016;**61**(9): 540-560. https://doi.org/10.1177/0706743716659417.
7. Malhi GS, Bassett D, Boyce P, et al. (2015) Royal Australian and New Zealand College of Psychiatrists clinical practice guidelines for mood disorders. *Aust N Z J Psychiatry* 49(12):1087-1206. https://doi.org/10.1177/0004867415617657.
8. Swift JK, Callahan JL. The impact of client treatment preferences on outcome: a meta-analysis. J Clin Psychol 2009;**65**(4): 368-381. https://doi.org/10.1002/jclp.20553.

**Figure S1.** Modulating effects of serum ghrelin on the associations of interleukin-6 (IL-6) tertiles with remission and relapse status.

^a^Interactive modulating effects of ghrelin on the associations of IL-6 with remission and relapse status were estimated using multinomial logistic regression; ^b^direct effects of IL-6 across tertiles within each ghrelin stratum, expressed as odds ratios (95% confidence intervals) [OR (95% CI)], were calculated using binary logistic regression, with the lowest tertile as the reference group, after adjustment for age, sex, monthly income, number of depressive episodes, current smoking, and scores on the Hospital Anxiety and Depression Scale–anxiety subscale and the Social and Occupational Functioning Assessment Scale for remission analyses; plus treatment step after 12 weeks and poor medication adherence after 12 weeks for relapse analyses.

| **Table S1.** Baseline characteristics by 12-week remission status in 1086 patients with depressive disorders. | | | | |
| --- | --- | --- | --- | --- |
|  | No remission (N = 596) | Remission (N = 490) | Statistical coefficients^a^ | P-value |
| **Socio-demographic characteristics** |  |  |  |  |
| Age, mean (SD) years | 55.9 (15.6) | 58.2 (13.9) | t=-2.601 | **0.009** |
| Sex, N (%) female | 411 (69.0) | 334 (68.2) | χ^2^=0.079 | 0.778 |
| Education, mean (SD) years | 9.1 (4.7) | 9.1 (4.9) | t=+0.065 | 0.948 |
| Marital status, N (%) unmarried | 192 (32.2) | 134 (27.3) | χ^2^=3.033 | 0.082 |
| Living alone, N (%) | 94 (15.8) | 73 (14.9) | χ^2^=0.158 | 0.691 |
| Unemployed status, N (%) | 186 (31.2) | 130 (26.5) | χ^2^=2.852 | 0.091 |
| Monthly income, N (%) <2,000 USD | 375 (62.9) | 273 (55.7) | χ^2^=5.801 | **0.016** |
| **Clinical characteristics** |  |  |  |  |
| Major depressive disorder, N (%) | 510 (85.6) | 415 (84.7) | χ^2^=0.164 | 0.686 |
| Melancholic feature, N (%) | 95 (15.9) | 67 (13.7) | χ^2^=1.088 | 0.297 |
| Atypical feature, N (%) | 35 (5.9) | 34 (6.9) | χ^2^=0.514 | 0.473 |
| Age at onset, mean (SD) years | 49.4 (17.1) | 52.8 (15.7) | t=-3.366 | **0.001** |
| Duration of illness, mean (SD) years | 6.7 (9.6) | 5.7 (9.0) | t=+1.808 | 0.071 |
| Number of depressive episodes, mean (SD) | 2.7 (5.6) | 1.8 (4.2) | t=+2.929 | **0.003** |
| Duration of present episode, mean (SD) months | 8.3 (12.0) | 6.4 (8.0) | t=+3.128 | **0.002** |
| Family history of depression, N (%) | 82 (13.8) | 76 (15.5) | χ^2^=0.664 | 0.415 |
| Number of physical disorders, mean (SD) | 1.6 (1.3) | 1.7 (1.2) | t=-1.212 | 0.226 |
| Body mass index, mean (SD) kg/m^2^ | 23.2 (3.3) | 23.2 (3.0) | t=+0.219 | 0.827 |
| Current smoking, N (%) | 80 (13.4) | 43 (8.8) | χ^2^=5.783 | **0.016** |
| **Assessment scales, mean (SD) scores** | |  |  |  |
| Hospital Anxiety & Depression Scale-depression subscale | 14.1 (3.9) | 13.1 (4.0) | t=+4.272 | **<0.001** |
| Hospital Anxiety & Depression Scale-anxiety subscale | 12.2 (4.0) | 11.3 (4.1) | t=+3.666 | **<0.001** |
| Social and Occupational Functioning Assessment Scale | 55.0 (7.6) | 57.1 (7.1) | t=-4.785 | **<0.001** |
| Alcohol Use Disorders Identification Test | 5.3 (9.2) | 5.4 (8.4) | t=-0.110 | 0.912 |
| ^a^Independent two sample t-test or χ^2^ tests, as appropriate.  Bold style denotes statistical significance (p-values<0.05). | | | | |

| **Table S2.** Baseline characteristics by relapse status after 12 week acute treatment in 710 patients with depressive disorders. | | | | |
| --- | --- | --- | --- | --- |
|  | No relapse (N=409) | Relapse (N=301) | Statistical coefficients | P-value |
| **Socio-demographic characteristics** |  |  |  |  |
| Age, mean (SD) years | 58.7 (13.9) | 56.8 (14.6) | t=+1.777 | 0.076 |
| Gender, N (%) female | 281 (68.7) | 212 (70.4) | χ^2^=0.244 | 0.621 |
| Education, mean (SD) years | 8.8 (4.9) | 9.3 (4.7) | t=-1.281 | 0.201 |
| Marital status, N (%) unmarried | 110 (26.9) | 83 (27.6) | χ^2^=0.040 | 0.841 |
| Living alone, N (%) | 63 (15.4) | 42 (14.0) | χ^2^=0.289 | 0.591 |
| Unemployed status, N (%) | 98 (24.0) | 87 (28.9) | χ^2^=2.199 | 0.138 |
| Monthly income, N (%) <2,000 USD | 242 (59.2) | 172 (57.1) | χ^2^=0.293 | 0.588 |
| **Clinical characteristics** |  |  |  |  |
| Major depressive disorder, N (%) | 347 (84.8) | 260 (86.4) | χ^2^=0.331 | 0.565 |
| Melancholic feature, N (%) | 65 (15.9) | 50 (16.6) | χ^2^=0.066 | 0.797 |
| Atypical feature, N (%) | 27 (6.6) | 20 (6.6) | χ^2^=0.001 | 0.982 |
| Age at onset, mean (SD) years | 53.1 (16.2) | 50.5 (15.9) | t=+2.095 | **0.036** |
| Duration of illness, mean (SD) years | 5.9 (8.9) | 6.6 (9.6) | t=-0.892 | 0.373 |
| Number of depressive episodes, mean (SD) | 1.7 (3.9) | 2.3 (4.7) | t=-1.893 | 0.059 |
| Duration of present episode, mean (SD) months | 7.1 (9.6) | 7.7 (10.6) | t=-0.765 | 0.445 |
| Family history of depression, N (%) | 57 (13.9) | 50 (16.6) | χ^2^=0.969 | 0.325 |
| Number of physical disorders, mean (SD) | 1.6 (0.5) | 1.5 (0.5) | t=+1.762 | 0.078 |
| Body mass index, mean (SD) kg/m^2^ | 23.3 (3.0) | 23.3 (3.1) | t=+0.187 | 0.852 |
| Current smoking, N (%) | 38 (9.3) | 33 (11.0) | χ^2^=0.589 | 0.463 |
| **Assessment scales**, mean (SD) scores |  |  |  |  |
| Hospital Anxiety & Depression Scale-depression subscale | 13.0 (4.0) | 13.9 (3.9) | t=-3.080 | **0.002** |
| Hospital Anxiety & Depression Scale-anxiety subscale | 10.8 (3.8) | 12.3 (3.9) | t=-4.936 | **<0.001** |
| Social and Occupational Functional Assessment Scale | 57.1 (7.5) | 55.9 (7.1) | t=+2.090 | **0.037** |
| Alcohol Use Disorders Identification Test | 4.7 (7.8) | 5.9 (9.7) | t=-1.855 | 0.064 |
| **Treatment related characteristics**, N (%) | |  |  |  |
| Treatment step after 12 week |  |  |  |  |
| Step 1 | 150 (36.7) | 101 (33.6) | χ^2^=8.314 | **0.040** |
| Step 2 | 147 (35.9) | 96 (31.9) |  |  |
| Step 3 | 86 (21.0) | 67 (22.3) |  |  |
| Step 4 | 26 (6.4) | 37 (12.3) |  |  |
| Poor medication adherence after 12 week | 141 (34.5) | 151 (50.2) | χ^2^=17.633 | **<0.001** |

| **Table S3.** Interactive effects of continuous serum interleukin-6 (IL-6) and ghrelin levels on remission: primary and sensitivity analyses across alternative modeling strategies. | | | | |
| --- | --- | --- | --- | --- |
| Model | N | Interaction  OR (95% CI) | Wald | P-value |
| 1 (primary): Continuous interaction adjusted for covariates identified in the main analysis | 1086 | 0.99 (0.99-1.00) | 1.239 | 0.266 |
| 2: Adjusted for a pre-specified set of clinically relevant covariates | 1086 | 0.99 (0.99-1.00) | 1.242 | 0.261 |
| 3: Log-transformed biomarkers | 1086 | 0.99 (0.99-1.00) | 1.373 | 0.243 |
| Models 1 and 3 were adjusted for age, sex, monthly income, number of depressive episodes, current smoking, and scores on the Hospital Anxiety and Depression Scale–anxiety subscale and the Social and Occupational Functioning Assessment Scale.  Model 2 was adjusted for a pre-specified set of clinically relevant covariates, including age, sex, number of depressive episodes, duration of the current episode, number of physical disorders, body mass index, current smoking, and scores on the Hospital Anxiety and Depression Scale–depression subscale. | | | | |
